# “Despite the Differences, We Were All the Same”. Group Cohesion in Diagnosis-Specific and Transdiagnostic CBT Groups for Anxiety and Depression: A Qualitative Study

**DOI:** 10.3390/ijerph18105324

**Published:** 2021-05-17

**Authors:** Anne Bryde Christensen, Signe Wahrén, Nina Reinholt, Stig Poulsen, Morten Hvenegaard, Erik Simonsen, Sidse Arnfred

**Affiliations:** 1 Research Unit for Psychotherapy & Psychopathology, Psychiatry West, Region Zealand, 4200 Slagelse, Denmark; nrei@regionsjaelland.dk (N.R.); sidar@regionsjaelland.dk (S.A.); 2 Department of Psychology, University of Copenhagen, 1353 København K, Denmark; signe.wahren@gmail.com (S.W.); stig.poulsen@psy.ku.dk (S.P.); 3 Mental Health Services, Capital Region of Denmark, 2200 København N, Denmark; morten.hvenegaard.pedersen@regionh.dk; 4 Psychiatric Research Unit, Region Zealand, 4200 Slagelse, Denmark; es@regionsjaelland.dk; 5 Department of Clinical Medicine, University of Copenhagen, 2200 København N, Denmark

**Keywords:** group psychotherapy, transdiagnostic therapy, cognitive behavioral therapy, group cohesion, anxiety, depression, mental health services

## Abstract

Group cohesion refers to a sense of belonging, mutual support and identification with other group members. Group cohesion has been associated with better outcomes, lower drop-out rates, more interpersonal support and better participation in psychotherapy. Nevertheless, the role of group cohesion in CBT has not yet received much attention. The rationale for delivering CBT in groups is that patients can model themselves through each other due to their similarities in symptoms. However, there has recently been a shift towards transdiagnostic CBT protocols, in which patients with varied diagnoses participate in the same groups. This shift challenges the rationale of delivering CBT in groups, and it is therefore highly important to understand if and how group cohesion develops in mixed diagnoses CBT groups. The current study used a qualitative comparative framework to investigate the patients’ experiences of group cohesion in diagnosis-specific versus transdiagnostic CBT groups. Twenty-three patients were interviewed with semi-structured interviews upon completion of the treatment. Participants had a primary diagnosis of MDD, panic disorder, agoraphobia or social anxiety disorder. A comparative thematic analysis was carried out. Three themes were found: *the move from differences to similarities*, *the role of group cohesion in group CBT* and *factors helpful and hindering to group cohesion.* Group cohesion developed across groups and was considered highly important in both diagnosis-specific and transdiagnostic CBT groups.

## 1. Introduction

Therapist alliance has been found to be an important, if not the most important, non-specific factor in individual therapy (CBT) [1]. However, in group therapy there are multiple systems of interpersonal relationships and interactions that are far more complex than a single dyadic relationship. Previous studies have found that the non-specific therapeutic processes for individual therapy and group therapy differ [2]. Clients participating in group therapy tend to place higher importance on the relationships with fellow group members compared to the therapist/group leader [2]. Group cohesion can be understood as the we-ness of the group; the qualities that bind the members together and foster liking, warmth, comfort and a sense of belonging. Group cohesion may also refer to interpersonal support, acceptance and esteem within the group [3].

Group cohesion has been understood as one of the key therapeutic processes in group psychotherapy and the foundation for all other group-related processes [4]. It has been defined in various ways, ranging from the ‘attractiveness of the group’ to a ‘unifying force’ and again to a more generic sense of support and belonging [2]. It has been argued that group cohesion is to group therapy what therapeutic alliance is to individual therapy [4]. In other words, in group therapy the relationships with fellow group members carries the therapeutic relationship, while the relationship with the therapist does the same in individual therapy. Some have argued that group cohesion is more complex than that, as it encompasses both the individual relationships to each of the other group members, as well as the relation to the group as a whole and the relationship with the group leader(s) [3]. This lack of clarity in definition has led to criticisms surrounding the construct itself. Rich descriptions of patients’ experiences of group cohesion may help define the construct further.

Group cohesion is understood as a key element in a range of therapies, i.e., existential and psychodynamic group psychotherapy where groups may consist of patients with a range of diagnoses or problems [4]. However, in cognitive behavioral therapy (CBT) group cohesion has not traditionally been considered a central aspect. CBT is oriented towards disorder-specific symptoms and an underlying belief that symptoms can be relieved through specific interventions such as cognitive restructuring and exposure. In other words, the focus of CBT is on specific factors and symptom relief [5] more so than on common factors.

Group cohesion is related to higher attendance rates, increased interpersonal support and augmentation of active participation in therapy [6,7,8,9]. A recent meta-analysis found group cohesion to be positively correlated to the outcome of psychotherapy [7]. The same meta-analysis found that the group–outcome relation was stronger in therapies that had an explicit focus on group processes compared with problem-focused groups such as CBT [7]. Furthermore, higher group cohesion has been related to positive psychotherapy outcomes and reduced drop-out rates [7,10]. Thus, CBT may benefit from utilizing group processes and focusing on the development of group cohesion.

CBT has been established as an efficacious treatment for major depressive disorder (MDD) and anxiety disorders, in both individual and group formats [11]. Due to an increasing number of clients seeking treatment for these disorders, there has been a shift towards group CBT as it is potentially more time-saving and cost-effective than individual treatment [12]. Furthermore, it has been argued that group CBT is advantageous over individual CBT due to the mutual support, modelling and a feeling of responsibility towards fellow group members [13]. Therefore, it is important to understand how group processes work in CBT. Much research has examined the treatment-specific factors in group CBT for anxiety and depression, i.e. homework adherence or reduction of depressive thoughts [14]. Furthermore, research into the non-specific factors such as therapist alliance and expectancy in group CBT has started to accumulate [15]. However, group cohesion has still not been studied extensively within CBT research.

The research into group cohesion in CBT for anxiety and depression has shown mixed results. Woody and Adessky (2002) found that group cohesion was not related to outcome in group CBT for social anxiety disorder, whilst Taube-Shift and colleagues [6] found that higher group cohesion was related to symptom reduction for patients with social anxiety disorder. In a study from 1974, Hand and colleagues found that in CBT groups for agoraphobia, patients from more cohesive groups had better results at follow-up; however, this effect was not present at the end of treatment [16]. Most recently, Norton et al. (2016) reported that group cohesion was associated with anxiety symptoms in the next session, albeit only in later sessions in the therapy course, which was transdiagnostic CBT for anxiety disorders [17]. Group cohesion has been established as a factor that is positively associated with outcome and lower drop-out rates in psychotherapy in general, but the results for CBT are mixed and the number of studies is low. Thus, there is a need for further studies in order to understand how group cohesion may or may not affect processes of change in CBT groups.

Recently, there has been a shift away from diagnosis-specific CBT protocols, towards transdiagnostic CBT (tCBT) protocols [17,18]. tCBT offers a range of advantages such as potentially treating comorbid disorders simultaneously, reducing waiting time in clinics and training therapists in just one manual. One such protocol is the Unified Protocol for treatment of emotional disorders (UP), which is designed to treat a range of emotional disorders such as anxiety disorders, MDD, Post Traumatic Stress Disorder (PTSD) and Obsessive Compulsive Disorder (OCD) by focusing on underlying neuroticism and emotion regulation rather than diagnosis-specific symptomatology. The UP has been found to be non-inferior to diagnosis-specific CBT for a range of anxiety disorders in individual therapy [18] and for social anxiety disorder, panic disorder/agoraphobia and MDD in group therapy [19]. One of the main arguments for delivering standard diagnosis-specific CBT in groups is that patients may experience normalization through similarities in symptoms and disorder-related experiences [20]. In group tCBT, patients may potentially have widely different symptomology and disorder-related behaviors and experiences. This challenges the arguments outlined in CBT research for delivering therapy in groups. Thus, in addition to the need for research on group cohesion in diagnosis-specific group CBT, there is a further need to investigate how group cohesion may be experienced in tCBT groups where patients have different psychopathological profiles and treatment needs.

Qualitative research provides the opportunity to explore phenomena in an explorative data driven manner and to access patients experiences with psychotherapy. Furthermore, qualitative studies can help us understand the ways that group cohesion may be related to other therapeutic processes and outcomes. The patients are not typically favorable towards specific processes or therapy types, the same way that researchers and clinicians are, and can therefore provide valuable descriptions of processes in a spontaneous and informative manner [21]. To the best of our knowledge, no previous qualitative studies focusing on the patients’ experiences of group cohesion in CBT or in transdiagnostic CBT exist.

The aim of the current study was to explore how patients in public Mental Health Services (MHS) experienced group cohesion in diagnosis-specific and transdiagnostic CBT groups, respectively. The research questions were:

*What is the role of group cohesion in CBT groups for anxiety and depression? Is group cohesion experienced differently by patients in mixed-diagnoses versus same-diagnosis groups? And if so, how?*

## 2. Materials and Methods

### 2.1. Context: TRACT-RCT

The current study was carried out within the TRACT-RCT [19], a multicenter, naturalistic, non-inferiority, randomized clinical trial. The therapy delivered in the TRACT-RCT was weekly group CBT over 14 sessions. The participants received either diagnosis-specific group CBT for either social anxiety disorder, panic disorder/agoraphobia or MDD, or transdiagnostic CBT in mixed-diagnoses groups through the Unified Protocol for emotional disorders (UP) [17]. All manuals were adapted to the context of group therapy in Danish MHS. The CBT groups consisted of patients with the same diagnosis. All transdiagnostic groups consisted of both patients with primary anxiety disorders and patients with a primary diagnosis of MDD.

The UP is one of the most well-established transdiagnostic CBT protocols, designed to apply across the emotional disorders, including anxiety disorders, depression and related disorders such as obsessive-compulsive disorder, somatoform disorders and post-traumatic stress disorder [22,23]. The protocol is an emotion-focused CBT intervention targeting neuroticism and resulting in maladaptive emotion regulation strategies [18]. Five core modules aim towards teaching adaptive emotion regulation strategies: (1) mindful emotion awareness, (2) cognitive flexibility, (3) countering emotional avoidance, (4) increasing awareness and tolerance of physical sensations related to emotions and (5) emotion-focused exposures. Three additional modules including motivation enhancement, psychoeducation on the adaptive nature of emotions and relapse prevention were also provided. We developed a Danish group version of the original individual UP protocol by Barlow et al. [18]. The original UP integrates CBT strategies (i.e., exposure, cognitive reappraisal) with other empirically supported strategies (i.e., mindfulness skills). In UP, however, these interventions focus on the patients’ emotional experience (e.g., anxiety) rather than the situation provoking the emotion itself (e.g., standing in a supermarket line).

### 2.2. Outpatient Treatments in Danish MHS

The treatment delivered in the trial was similar to the standard treatment offered in Danish outpatient clinics, in terms of intensity and duration. The clinics are publicly financed secondary services for individuals suffering from moderate to severe non-psychotic disorders. The clinics offer a standardized, time-restricted treatment format [24] which includes psychotherapy, medical consultations and a session for relatives.

### 2.3. Participants

The current study included 23 patients from four psychiatric outpatient clinics in Denmark. All of the patients had participated in the TRACT-RCT [19]. Participants in the TRACT-RCT were contacted by telephone upon completion of the treatment and asked if they wished to participate in an interview regarding their experiences with therapy. The sampling was targeted so that there would be an equal division of patients from the transdiagnostic therapy groups and the diagnosis-specific groups, as well as an equal division of patients with a primary anxiety diagnosis and a primary diagnosis of MDD. Ultimately, the sample consisted of 12 patients who had received treatment in transdiagnostic groups and 11 who had received treatment in diagnosis-specific groups. The participants were recruited from 17 different therapy groups and represented all four types of therapy groups:

1. TCBT—Unified Protocol (UP) for MDD, agoraphobia, panic disorder and social phobia mixed group
2. CBT depression for MDD
3. CBT social anxiety for social anxiety disorder
4. CBT panic for panic disorder and agoraphobia

See Table 1 for patient characteristics. All of the included patients had completed the 14-week therapy course at the time of the interview. Completion was defined as having participated in 8+ sessions and not having discontinued treatment.

### 2.4. Ethical Considerations

Participation was voluntary. All participants gave written informed consent. The data were anonymized. The original transcripts were only accessed by ABC and three research assistants who transcribed the audio files. All participants were debriefed by ABC upon completion of the interview.

### 2.5. Data Collection

The data were collected through semi-structured interviews. An interview guide was designed to explore several aspects of the patients’ experiences of psychotherapy, with group cohesion being just one of them. See Table 2 for the interview guide.

The interviews were carried out in the clinics throughout 2018 and in January of 2019. The participants were interviewed once and the interviews lasted approximately one hour in duration. All interviews were audiotaped and transcribed verbatim.

### 2.6. Data Analysis

The data analysis was a thematic analysis, inspired by the step-by-step approach taken by Braun and Clarke (2006) [25]. This analysis method is atheoretical and well-suited for describing and finding themes across large qualitative datasets such as this one. In the current study we followed all the six steps of analysis identified by Braun and Clarke [25]. Furthermore, we added two additional steps in order to strengthen the analysis and to add the comparative component. The analysis was carried out with NVivo software. The analysis consisted of the steps detailed below:

1. All of the transcripts were read thoroughly in order for the first author to familiarize herself with the data.
2. Two full transcripts were coded on a sentence-to-sentence basis by the first author and the second author separately.
3. The first and second author held a meeting in which they went over every single code, discussing and debating in order to reach consensus if any discrepancies were present.
4. Step 2 + 3 were repeated twice, meaning that 6 full transcripts were consensus coded (26%), on the sixth interview, only 5 codes differed between the coders, indicating very high consensus in the coding. The first author coded the remaining transcripts.
5. A list of group cohesion-related codes was created, and all of the material labelled with those codes was extracted from the full dataset. The group cohesion-related quotes were chosen by going through the coding set and, firstly, choosing all the codes that had the word ‘group’ in it, i.e., group dynamics, the group, group members etc. Secondly, ABC went through all remaining codes and read the related material; all codes that had group-related content were included, i.e., the breaks, normalization and similarities and differences.
6. The codes were revisited in the search for overarching themes. An inductive approach was taken, meaning that no theory was driving the process.
7. The dataset was divided into two: (a) the data belonging to patients who had been in diagnosis-specific groups and (b) the patients who had been in mixed-diagnoses groups. This was completed in order to detect if there were differences between the groups within each theme.
8. Themes were named and the model of the group cohesion process was defined.

## 3. Results

**Figure 1:**
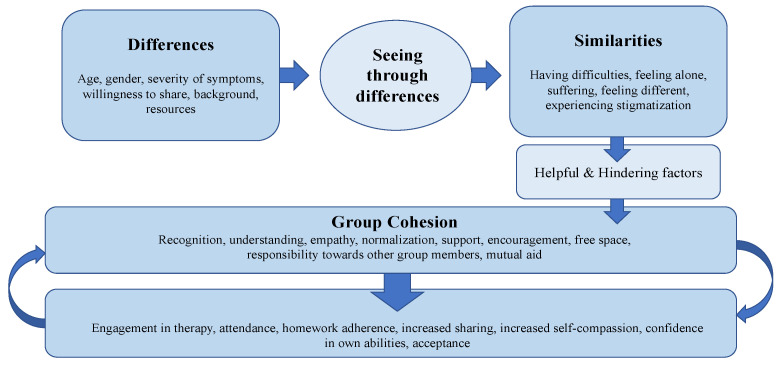
Group cohesion model.

The analysis revealed three emerging main themes: *the move from differences to similarities*, *the role of group cohesion in group CBT* and *factors helpful and hindering to group cohesion*. The themes were linked in a chronological order and presented with verbatim quotes and a model of the process of the development of group cohesion. A model of the development of group cohesion was created based on these results (Figure 1).

### 3.1. Theme 1: From Differences to Similarities

The interviews revealed that the patients described differences between themselves and the other group members in the first sessions of therapy. There was an overarching consensus that the noticeable differences in the group were variables such as age, gender, occupation, severity of symptoms and ‘life stage’:

I: How did you experience the other group members?

Caroline, UP group: Well, we were at different places in life. Some had not yet started education. Some of us had been on the labour market for many years. Some could not work and had tried to get early retirement. So, we were a very mixed group. And then we were only women... but our group worked. Despite all these differences. Because it was not those differences in our private lives, in that way, that was the reason why we were here. We just had, many of us, the same symptoms… and fundamentally for most of us, those were about the same things.

I: How did you experience the other group members?

Cecilia, Panic-disorder group: It was very varied, in terms of, how burdened they were by their symptoms, I think. And that was actually pretty positive, that we weren’t all equally well or unwell. Because, then you could say, that you can kind of put yourself into perspective in relation to them, like ‘that person is that well and that person has come that far’, I mean not in a negative way, but, more... like it gave a sense of lifting each other up, and that you could each see positive things in the others, so you got a sense that ‘oh well then I can also do it’ (...) There were probably more differences than similarities. Because there was differences in terms of ehm... Well, age, gender and where people where on all levels, it seemed. But it wasn’t something I had really thought that much about (...) again, I think it is positive, because you can put it all into perspective and it gives you a feeling that ‘oh well we are not just all the same’ but ehm... it’s all kinds of people, who feels this way in each their way, and yeah, I thought that was positive.

Caroline pointed to differences in occupation and ‘life stage’, and Cecilia also pointed to the group members being different in terms of age, gender and ‘where they were on all levels’. All these factors were commonly pointed to, across the interviews, as differences. Both of the women highlighted that the detected differences were not seen as a problem, and Cecilia went on to explain how the diversity could actually work as a motivator, by showing the different ‘stages’ in an illness. Most of the patients described how the differences between themselves and others were the first things they registered when walking into the first group session, which explains why the visible differences such as age and gender were among the most frequently mentioned differences. The majority of the patients described how the initial impressions of many differences were quickly replaced by a sense of detecting similarities:

Katrine, UP group: It was a really good group. We have agreed, that we will meet up in two weeks time, in order to keep it maintained… or to support one another. I mean, we were wildly different all of us. I mean, really wildly different and different ages and did completely different things and also had different diagnoses, but we worked pretty well together.

I: Yes?

Katrine, UP group: I mean, we could recognise ourselves in each other and in the problems that we have had, even across diagnoses.

I: So it wasn’t something that stood in the way?

Katrine, UP group: *Not at all.*

Katrine described how the differences were not experienced as a problem, because the patients recognized themselves and their problems in each other, despite diagnostic differences. Most of the interviewed patients described starting to see through differences already in the first or second session. A small number of patients said that it took longer:

Karen, UP-group: At one point, one of the others was like ‘why do I have to sit with those who have anxiety? I have a depression’ and I had both diagnoses and some only had the anxiety diagnosis… and all of a sudden it occurred to her, that the things that I said, when I described how the anxiety affected me, then she said ‘that is exactly how I feel and God, that is the same. And that’s when we figured, that, that is why they are trying to put these two things together in the treatment, because it is so similar in structure. And that’s when it suddenly occurred to her, I think it was the fifth or sixth session, when she saw the light, like ‘wow, it is the same’.

This extract shows that there were different routes to the experience of similarity. The patients did not need to share diagnoses or specific symptoms, but realized that the way their problems affected them and their lives was similar. It also pinpoints the process of seeing through differences and focusing on the characteristics that connected group members, rather than those that separated them. The process of seeing through initial differences appeared to be key in establishing a sense of connection to the group and enforcing a belief in the treatment. The experience of realizing similarities brought a feeling of safety and comfort to the patients, and that made them engage in the therapy in new ways.

### 3.2. Theme 2: The Role of Cohesion in Group CBT

Having seen through the differences that separated themselves from the others, most of the patients went on to explain how the discovery of similarities sparked group cohesion through factors such as universality and mutual understanding. In the following extracts, Sara and Eva explained what group cohesion added to their therapy experience:

Sara, Depression group: It gave me, how do I explain it? You feel like you are all alone in the world somehow, prior to coming. And I found that difficult. I mean... it feels like a taboo, when you speak to others, friends as well... When you speak to friends, there isn’t really anyone to mirror yourself in. So, when you start in this group, even though I was one of the youngest, it was still really nice to have these people here to mirror yourself through. And we could talk together, and we were in the same place. And knew the problems, even though the causes were different. And I found that incredibly comforting. I thought it was great and it gave me a lot, this thing about mirroring yourself in someone else. Even though it is negative, it is just really nice to know that you are not all alone in it.

Eva, UP group: In the beginning, we were all insecure. But they were engaged and worked as much on it as I did. There was a will to want this. The youngest was 21 and I am 52 and I thought ‘wow do we have anything in common?’ It didn’t feel unnatural despite that. And many of them were students or academics and I am a musician. And have, of course, sometimes a different understanding of the world. It is interesting to hear what kind of things you can be battling. We had different problems, but basically, anxiety and depression and so on, it was very similar, but we came with different backgrounds and things that had triggered our problem. The fact that we were doing as poorly as we were meant that we had a lot of practical problems too. When you don’t have peace to just get well. Because there are so many practical things you have to fight. That we had in common, a lot. The system around us. There was a pressure. From outside. We are fighting basic survival stuff.

Sara explained how feelings of stigma and taboos around mental health were relieved by meeting others who suffered. Likewise, Eva explained that despite differences between group members, suffering, distress and struggling with ‘the system’ was the same for all. Both women contrasted being inside the group against being in the outside world, underlining a strong sense of belonging to the group. They both went on to explain how just being able to meet others who have similar problems had removed feelings of loneliness and provided a place where one could talk about suffering with others who understand. We understood these types of statements as manifestations of group cohesion. Group cohesion made patients feel a responsibility towards the group that in the end pushed their own improvement forward.

Peter, Depression group: I had felt very alone in it. Alone with my struggles (prior to beginning in the group). Even though I am not directly comparable with everyone, then... the story is the same, but the details are different. I think, when I was out here in sessions on my own, I felt very alone in it all. But being put in a group also creates group pressure somehow, that makes it easier to get homework and tasks done, I would say.

I: Because?

Peter, Depression group: Because, then there is a whole group you can disappoint all of a sudden. And that is kind of positive. A little bit of peer pressure. Whatever works, you know?

I: Okay, and being together about it, not being alone, how does that impact you?

Peter, Depression group: It does a lot for your sense of self-worth. Seeing that it is normal, that other people fight it too. I am not a uniquely bad human being. There are tons of people who feel this way, and they are not bad people, therefore... and so on. I think about it as in, your shoes become less heavy, it is like there are springs in your step when you walk. That you are no longer dragging yourself, all the time.

Peter explained how group cohesion created a sense of responsibility towards the group that motivated him to do his homework assignments by adding ‘*a bit of peer pressure*’, and how he viewed that as a positive thing. He also went on to explain how group mediated processes have impacted his feeling of self-worth directly, by making him see that others who feel like him are not bad people. He metaphorically summarized how that eased the burden of having depression. Patients often described ‘*lifting the burden together*’ and helping each other through mutual responsibility, respect and support towards the group.

### 3.3. Theme 3: Factors Helpful and Hindering to Group Cohesion

All of the interviewed patients described group-related processes such as normalization, support, belonging and mutual aid as being important, if not the most important elements of treatment. We understand these constructs together, as a manifestation of group cohesion. Several of the patients highlighted three distinct factors that impacted this process in either a positive or negative direction: (a) the motivation of the other group members, (b) the breaks, which were seen as helpful factors in aiding group cohesion and (c) group members who were perceived as unable to share, which was seen as a hindering factor. Group cohesion appeared to form despite the issue of members who did not open up in the group (through the help of the therapist); however, this tended to cause frustration and insecurity. The motivation or readiness to change in the other group members was highlighted as a helpful factor as it created the sense of collaboration and lifting together in the group:

Tina, UP group: We had a lot of the same ways of thinking and ways of seeing the world. We were all there because we had some problems that we couldn’t solve on our own, and we wanted help and we had a wish for things to get better. We wanted to work and make things better. I feel like that really tied us together.

Several patients mentioned the 10 min breaks in the middle of the therapy session as a factor that aided group cohesion:

Jonathan, Depression group: The biggest help... has actually perhaps been, even though I didn’t say that much, just meeting the other and hearing about and listening to their stories. I think, the breaks were the biggest help. It was a joy to meet likeminded people.

The breaks were highlighted by patients because they provided a space for free talk and being ‘just human’ with one another. Several patients explained how the breaks gave group members a chance to bond over aspects other than illness and made them connect on a different level. The analysis also detected one commonly mentioned factor that was experienced as hindering the process of developing a sense of group cohesion:

Peter, Depression group: There was especially one person, that I am thinking of now, who weren’t really there. Or who was very shy or holding back, had a lot of barriers. I actually felt like it was hindering to creating an optimal good dynamic. I do think the Clara (the therapist) was really good, even though this person didn’t say anything, she kept making openings for the person... and it took the time it took, but I feel like, when she did that, it helped a lot on the dynamic, when everyone shared, it was easier to share more. At least it made it feel safer. That there wasn’t a stranger in the corner who didn’t say anything.

This extract illustrates how insecurity and safety were compromised when one or more members of the group were perceived as unable to share. While this factor was echoed by others throughout the material, it was not an overarching theme.

## 4. Discussion

The current study aimed to explore the role of group cohesion in patients’ experiences of group CBT for anxiety and depression in an MHS context. Furthermore, the study set out to compare data from 12 patients who had participated in mixed-diagnoses, transdiagnostic CBT groups with the data from 11 patients who had participated in diagnosis-specific group CBT for MDD, social anxiety disorder or panic disorder/agoraphobia [26]. When discussing the results in the following sections, we define group cohesion as a feeling of belonging to the group and being mutually supported and supportive of the group.

The results showed that group cohesion and other group-related processes were experienced as major, if not the most important, factors in the group therapy, across treatment types. Patients tended to notice how they were different from other group members in the beginning, then quickly started to see through the differences and noticed the similarities that bound them together; therefore, this process laid the foundation for group cohesion. Group cohesion was developed through a range of group-related processes such as normalization, anti-stigmatization, mirroring, support, encouragement and understanding. The sense of belonging to the group and being mutually supportive and supported by the group led to a higher motivation to attend the therapy and to do homework assignments, a larger willingness to share in the group and gave renewed hope for the future, according to the patients. This was consistent with the assumptions made about the positive features of group cohesion in previous studies [6].

Surprisingly, the patients in the transdiagnostic groups did not talk about diagnostic differences. They tended to point to global constructs when explaining how they bonded, such as suffering, feeling alone or experiencing stigmatization in the outside world. It may be that the broader focus in the UP manual helped this process of recognition across diagnoses. The UP manual does not focus on disorder-specific symptoms or psychopathological categories. Instead, both depression and anxiety disorders are seen as expressions of underlying neuroticism, which makes patients at risk of experiencing ‘negative emotions’ as uncontrollable, dangerous and overwhelming. This experience leads to the development of non-adaptive coping strategies that reinforce the same negative emotions the patient was trying to avoid in the first place [17]. The manual is designed to treat patients with different symptomology within the same groups. Accordingly, this treatment perhaps embraced differences between patients to a higher degree and explicitly helped the patients to understand and verbalize the different symptomologies as different expressions of the same thing. This was illustrated in the language that the patients from the UP groups adopted, e.g., “*depression and anxiety, at the core, they are the same*”. It may also be that the therapists who delivered this therapy were highly aware of zooming in on similarities between patients to create cohesion despite diagnostic differences.

Thus, the development of group cohesion appeared to happen in both treatment groups, and the feeling of belonging to the group was highlighted as vastly important by all of the interviewed patients. In other words, group cohesion was experienced as a core healing factor in the therapy, as well as a factor that motivated and assisted patients in attending the therapy, engaging in the therapy and adopting the manual-specific techniques. These positive consequences appeared to strengthen the sense of cohesiveness in the group.

Several previous qualitative studies have found that patients highlighted group mediated factors as positive and essential to the experience of group CBT for anger management, schizophrenia, auditory hallucinations, eating disorders and hallucinations [27,28,29,30]. The role of group mediated processes such as group cohesion, universality, mutual aid, interpersonal learning and renewed hope have been well described in the psychotherapy literature by Yalom [4]. However, these concepts are derived from psychotherapies with fundamentally different theoretical underpinnings than CBT. Nevertheless, although the group is not recognized as an independent healing constituency in the CBT literature [20], but rather as a factor that may help optimize specific processes and outcomes, it appears to be experienced as a healing constituency on its own, by the patients in the current study. This is consistent with previous literature that found that clients in group therapy tended to see the other group members as at least as beneficial as the group leaders [2].

Group cohesion appeared to be connected to the patients’ engagement in the therapy. This is consistent with research demonstrating the effect of group cohesion on outcome and in reducing drop-out rates [3,7,8,9,10]. Thus, it appears that group cohesion can impact the outcome through two routes: (a) a direct impact of the positive factors associated with group cohesion, and (b) the indirect impact of group cohesion on therapy engagement and compliance.

Norton et al. (2016) found that group cohesion impacted the outcome in the next session, but only in later sessions. This may be an indication that group cohesion takes time to form, but when it does it can impact outcomes [18]. This is consistent with the findings of the current study, in which patients explained that it took some time to see through the differences and feel a sense of belonging to the group. There were many individual differences in terms of how long it took to feel belonging to the group, although most of the participants expressed already starting to see similarities in the first session.

The current study also found several factors to be helpful in the creation of group cohesion. The first factor was the motivation or readiness to change in the other group members. This factor is similar to Yalom’s factors, *mutual aid* and *imitative behavior*, and can be seen as one of the strong benefits of group therapy [4].

In the current study, a hindering factor was brought to light, i.e., that patients who appeared unable to share created a breach in the development of group cohesion and made group members feel unsafe and as if “*there was a stranger in the corner*”. This illustrated how patients experienced the group as a place where all members must invest for a safe environment to form and the dynamics to be optimal. One may look to the model described by Poulsen (2004) in which two key concepts are thought to be central for the group’s developmental processes, namely ‘de-privatization’ and ‘emotional presence’. The model describes how recognition in fellow group members initiates the process of de-privatization through which the patient will open up due to the normalization and anti-stigmatization that recognition provides. On the contrary, if the patient does not experience recognition, the de-privatization process will not initiate. The de-privatization process is largely dependent on the emotional presence of the group members and the group climate [31]. This could explain why it may have felt like a hinderance when others did not share.

Although the de-privatization model was based on a qualitative study of short-term psychodynamic therapy, it appears to fit well with the concepts identified in the current CBT study. This highlights that although CBT research has not traditionally been interested in group processes, they do play a role in group CBT and they look much the same as in other, fundamentally different types of therapy. Thus, we recommend that future research focuses on the role of common factors (other than alliance) in CBT.

Interestingly, several patients described the breaks, as well as the time before the session started and after the session ended, as helpful for creating group cohesion. These time slots were described as a time where the patients had space to talk about other aspects of their lives and bond on a different level. It may well be that the highly structured and content-loaded approach of CBT did not leave much space for this type of interaction to take place within the therapy room, and that, accordingly, it was particularly important for the patients to seek out this type of interaction in other ways, i.e., in the breaks.

### Limitations and Strengths

The current study was a qualitative interview study based on 23 interviews. While this type of study is useful in helping us understand processes and provides deep descriptions, the results cannot be generalized to broader populations of contexts, without further studies.

No patient who had dropped out of the treatment was interviewed. It is highly possible that they may have provided different perspectives on the development and feeling of group cohesion. We did not collect information on minority status, which may have been useful in making sure minority groups were heard, and in understanding whether their perspectives were different.

The sample in the current study was large and strategically selected. The participants represented a range of demographics, outpatient clinics, diagnoses and treatment types. The findings were clear despite the varied characteristics of the participants strengthening the results.

## 5. Conclusions

The research into how group cohesion impacts group CBT and transdiagnostic group CBT has been sparse and the results have been inconclusive. The current study found that patients, when describing their experience, highlighted a number of group-related factors, that can be understood as group cohesion. This was described as essential as well as an aiding tool for adherence and engagement in therapy. Furthermore, the current study recommends that group therapists in both diagnosis-specific and transdiagnostic groups focus on verbalizing similarities between patients, not just in terms of symptoms, but in terms of more global constructs such as distress. This is thought to aid the process of creating group cohesion.

## Figures and Tables

**Table 1:** Participants Characteristics.

Treatment Type	Pseudonym	Sex	Age	Primary Diagnosis	Comorbid Diagnosis
tCBT	Marcus	M	28	MDD	-
	Eva	F	55	MDD	-
	Niels	M	35	MDD	-
	Emma	F	34	MDD	-
	Laura	F	43	MDD	-
	Katrine	F	32	SA	ADHD
	Simone	F	65	PD	-
	Caroline	F	42	MDD	-
	Karen	F	53	MDD	PD, AGO
	Tina	F	25	PD	AGO
	Victoria	F	54	PD	AGO, SA MDD, GA
	Julie	F	28	SA	MDD
CBT	Michael	M	28	PD	AGO
	Josephine	F	22	SA	OCD, GA
	Simon	M	54	MDD	PD
	Barbara	F	31	PD	AGO
	Jonathan	M	49	MDD	SA
	Peter	M	41	MDD	SA
	Michelle	F	26	MDD	PD
	Sara	F	25	MDD	-
	David	M	30	SA	PD
	Cecilia	F	38	PD	AGO
	Kristine	F	37	SA	ADHD, GA

Note: PD = panic disorder, AGO = agoraphobia, SA = social anxiety disorders, GA = generalized anxiety disorder, ADHD = attention deficit/hyperactivity disorder.

**Table 2:** Interview Guide.

Topic	Examples of Questions
1. Open talk	Tell me about your therapy course, whatever comes to mind.
2. Expectations	What were your expectations prior to starting in the group? Were these expectations met?
3. Group	How did it feel to be in this group? How were the other group members?
4. Important moments	Were there any specific moments from the therapy that you remember especially well?
5. The therapists	How did you find the therapists? Were there any moments with the therapists you remember particularly well?
6. Change	Do you feel different now compared to when you started? If so, how? Why do you think that is?
7. The therapy	What was the biggest help for you? Did anything happen that was negative for you?
8. Manual specific factors	Where there any specific techniques you found especially helpful?
9. The end	Do you feel like I have a good understanding of your experience? Have we missed anything that was important for you?

## Data Availability

No data available.
